# Supplementary material for: Differential impact of the COVID-19 pandemic on laboratory reporting of norovirus and Campylobacter in England: A modelling approach
Source: PLoS One. 2021 Aug 25;16(8):e0256638. doi: 10.1371/journal.pone.0256638 (PMC8386829; doi:10.1371/journal.pone.0256638)
Supplement: Attachment — Submitted filename: Response to Reviewers.docx [file pone.0256638.s001.docx]

We appreciate the time and effort that the reviewers have dedicated to providing their valuable feedback. We have been able to incorporate changes to the analysis and the manuscript to reflect the comments provided.

Here is a point-by-point response to the reviewers’ comments and concerns.

Comments from Reviewer 1

- **Comment 1**: *I would recommend the authors add some more explanations and also describe the contribution of this work in the introduction section*

**Response:** Even though, the contribution of the study is described in the discussion, I agree that it is good to mention it more in the introduction too. The highlighted text was added to the introduction.

Changes in the manuscript (Introduction, lines 123-127):

… To the best of our knowledge, this is the first study to compare, both quantitative and qualitative differences in the pandemic’s impact on the number of norovirus and *Campylobacter* reports. Additionally, the analysis provides insights into how to account for the pandemic in detection algorithms and predictive models used more broadly in public health.

- **Comment 2** (Page 10 of 21): *“The conditional distribution was chosen as negative binomial to account for over-dispersion, and the link function was logarithmic.” How the conditional distribution is selected any reference or source please provide details.*

**Response:** A negative binomial model is a standard approach to dealing with over-dispersion: for background context and to guide the reader we have inserted reference 15 was added (Materials and methods, line 193).

- **Comment 3** (Page 13 of 21): *“In particular, changes in the stringency index were associated with a reduction of ~7% (CI 2% - 6%)” check these confidence limits.*

**Response:** Thank you for pointing this out – yes, this was indeed a typo. These values have further changed since the second model was updated in response to Referee 2 (Results, lines 249-254).

- **Comment 4** (Table 3): *The values of Sars-cov-2 tests is not correct for norovirus.*

**Response:** The model was updated so this table has now changed.

- **Comment 5** (Table 3): *Construct a table that lists the character shorthand.*

**Response:** We have modified the caption to Table 3 to reflect the shorthand, since this seems to be the most parsimonious way to represent this information.

- **Comment 6** (Figure 1): *From Figure 1 it seems that the Actual data is following downward trend while the predicted data trend is upward. Is this a real picture of the data or correct modelling?*

**Response:** The Figure 1 (now Fig. 2) shows what would have happened had there been no pandemic. While norovirus in England tends to have winter seasonality and is expected to decrease in spring, *Campylobacter* reports tend to increase in spring. However, due to the pandemic, this was disrupted and so it is expected that the prediction (what would have happened) and the actual data (what happened) will look different.

Changes in the manuscript (Results section, line 228):

As illustrated by Fig 2, this is likely because the effect was too short to be detected with higher level of significance.

Notes under Fig 2 (lines 239-242): The figure displays the model’s prediction (Fitted) and the actual number of weekly reports (Actual). The section with the ribbon, i.e. confidence interval, highlights what was expected in the absence of a pandemic.

Comments from Reviewer 2

- **Comment 1** (Figure 2): *In the figure, norovirus cases reduce from ~200 per week pre-COVID to <20 cases per week post COVID, which is a >90% reduction. Why are the estimates presented in Table 2 significantly lower, even for delta=1?*

**Response:** We agree this needs clarification. The pandemic arrived in the UK at the time of the year when norovirus usually decreases. Since our modelling accounts for the seasonality, some decrease is expected and shouldn’t necessarily be attributed to the pandemic. Similarly, this applies to the increasing air temperature (weaker for Campylobacter) and effect of easter holidays (dip in reporting). Also, the model considers the autoregressive nature of the weekly reporting. Therefore, the observed difference between the lines does not equal the estimated impact of the pandemic. On top of that, the actual difference between the Actual and Fitted lines in the pandemic period is ~86% and since the model’s Mean Absolute Error (MAE) on non-pandemic period is ~20 reports the estimates we provide make sense. Even the model assuming the start of the pandemic on week 13 showed point estimate of 62% (CI 53% - 70%); confirming the stability of the estimate.

Changes to the manuscript (Results, lines 265 - 280):

To make the models easier to interpret and compare, an impact variable was added to the second model to capture residual effects of the pandemic unexplained by the proxies, i.e. number of Sars-CoV-2 tests conducted and stringency index. The section comparing the models now discusses total effect estimated by both models. This new addition widened the estimates, and these were updated in the abstract and the discussion. However, these still might be conservative and so this has been added to the limitations.

(Discussion, line 350)

Modelling both pathogens brings many challenges; … Considering all of these challenges, our estimates might be conservative.

- **Comment 2:** *Similarly, for Campylobacter the decrease is from ~1,000 to 250 which corresponds to a 75% reduction. But again, the estimates are lower.*

**Response:**  Similarly, to the response above, there is a lot of uncertainty, e.g. seasonality can differ by region (see [Louis et al., 2005](https://www.ncbi.nlm.nih.gov/pmc/articles/PMC544220/)). In our paper, the estimate is provided as a mean value over the 32 weeks. The estimated impact at the beginning was between 38% – 55% but then it decreased exponentially. The actual difference between the lines is ~34% during the pandemic and the MAE in the period before the pandemic was ~76 reports. Clarifications were added to the manuscript (see highlights below).

Changes in the manuscript (Materials and methods, lines 165-170):

… For example, level shift is the same on the first as well as the fifth or tenth week, while for transient shift, the highest impact is assumed at the beginning, but decreases exponentially with time, e.g. (δ = 0.85) in the second week but (δ = 0.52) in the fifth week (see Fig. 1). The estimated coefficient of the indicator is then a relative change in the weekly laboratory reports of a given pathogen, considering the other variables and effects in the model such as air temperature, seasonality and autoregression.

- **Comment 3:** *I agree with the authors assessment that delta=1 for norovirus and approximately 0.85 for Campylobacter but they do not provide any justification for the later value. You could argue, for example, that the reduction in Campylobacter halves over 4 weeks and 0.85^4=0.52.*

**Response:**  The reduction for Campylobacter indeed halves over 4 weeks. As described in the methods, we compare level and transient shifts. The transient shift represents exponential decay. To clarify this more in the paper, a plot visualising the individual effects was added as well as specific examples in the text.

Changes in the manuscript (Results, lines 224-228):

The decrease in *Campylobacter* reports was better described by transient shift (δ = .85). The estimated impact was ~46% (CI 38% - 55%) on week 12, ~39% (32% - 47%) on week 13 and so on; the mean weekly reduction across weeks 12 and 43 was ~9% (CI 8% - 11%). This decrease was statistically significant at the 10% but not the 5% level (p = 0.075).

- **Comment 4:** *It is a bit of shame that delta is not estimated from the data. Fitting a transfer function model would be one way to do this - see for example chapter 13 of “Time Series Analysis” by Box, Jenkins , Reinsel and Ljung (a pdf version of the book is available for free online)*

**Response:**  As the paper aims to demonstrate not only quantitative, but also qualitative difference between the impacts of the pandemic on norovirus and Campylobacter, I think the current format of the analysis achieves this better, i.e. it allows for easier communication of the message – Norovirus is more similar to Sars-CoV-2 than *Campylobacter* and so the reduction in norovirus is related to the actual measures, not only changes to the health seeking behaviour. It is our feeling that a more detailed analysis along the constructive lines suggested is beyond the scope of our paper.
