# Supplementary material for: Differential impact of the COVID-19 pandemic on laboratory reporting of norovirus and Campylobacter in England: A modelling approach
Source: PLoS One. 2021 Aug 25;16(8):e0256638. doi: 10.1371/journal.pone.0256638 (PMC8386829; doi:10.1371/journal.pone.0256638)
Supplement: Attachment — Submitted filename: Response to Reviewers.docx [file pone.0256638.s002.docx]

We appreciate the extra effort that the reviewers have dedicated to providing additional feedback. We have incorporated changes to the manuscript and one of the figures to reflect the comments provided.

Here is a point-by-point response to the comments.

Comments from Reviewer 2

*I would like to thank the authors for addressing my comments. In my view the paper is acceptable for publication. I have one question and a suggestion.*

- **Question**: *I was unsure how to interpret the norovirus shift parameter estimate in the new Table 3. I imagined exponentiating this parameter would give me the measure of impact but it doesn't (exp(-0.792)=0.45 but the reported impact is 59%). Is this an error or how should this parameter be interpreted?*

**Response:** Thank you for spotting this, it needs clarification in the text. Briefly, since three variables represent the impact in the second model, the estimate is a sum of these three variables giving us ~59% decrease.

Specifically, all coefficients are transformed by exponentiating and subtracting one – *(exp(coefficient) – 1) * 100*. So the shift is then *(exp(-0.792) – 1) * 100* ~ 55%. In the second model, we have two more variables indicating impact, which are added to the shift. As stringency index and the number of conducted Sars-CoV-2 tests vary over time, we need to multiply the coefficients by the actual values of the respective variable. Additionally, we only consider the period between weeks 12 and 43 to match the timeline of the shift variable. For norovirus, both the conducted tests and stringency index give us a decrease of approximately 2%, which is 4% in total and 55% + 4 % gives 59%. The simpler overall model represents the pandemic only with the shift indicator where *(exp(-0.880) -1) *100* ~59%.

Changes in the manuscript (Results, lines 271-281):

… A simpler (overall) model might have underestimated the impact as the total decrease in norovirus reports determined by the second (specific trends) model has wider confidence intervals ~59% (CI 47% - 79%). On the other hand, the point estimates of both the Overall Impact and Specific Trends models were ~59%. Similarly, the total mean reduction of Campylobacter reports estimated by the Specific Trends model was between ~11% (CI 8% - 17%), which is higher than the simpler Overall Impact model ~9% (8% - 11%). Note that the impact estimate from the Specific Trends models is a sum of all the pandemic related estimates, i.e. conducted Sars-CoV-2 tests, stringency index and the shift determined by the overall impact model.

- **Suggestion**: *Given that seasonality is so important in this analysis it might be worth illustrating it somehow - e.g. by promoting the weekly time series figures from the supplementary information to the main text.*

**Response:** Thank you for the suggestion. Figure 2 now includes the previous season (2018/19), showing two years in total, and the seasonality is visible there.
